# Identifying meaningful aspects of health and concepts of interest for assessment in systemic lupus erythematosus: implications for digital clinical measure development

**DOI:** 10.1186/s41687-024-00832-7

**Published:** 2024-12-24

**Authors:** Paul Kamudoni, Kate Lyden, Oliver Günther, Vikas Jaitely, Thiago Dantas Araujo, Erica Spies, Josephine Park, Erik Thomas, Joy Buie, Jennifer M. Blankenship, Laurent Arnaud

**Affiliations:** 1 Clinical Measurement Sciences, The Healthcare Business of Merck KGaA, Darmstadt, Germany; 2 VivoSense, Newport Coast, CA USA; 3 EMD Serono, Billerica, MA USA; 4 Lupus Foundation of America, Washington, DC USA; 5 Department of Rheumatology, Hôpitaux Universitaires de Strasbourg, INSERM UMR-S 1109, Centre National de Référence des Maladies Auto-immunes Systémiques Rares (RESO), Strasbourg, France

## Abstract

**Objectives:**

Systemic lupus erythematosus (SLE) is a chronic autoimmune disease with heterogeneous clinical manifestations which significantly impacts the daily lives of patients. Herein, we aimed to (i) investigate patients’ perspectives on and experience with SLE; (ii) identify meaningful aspects of health (MAHs) and concepts of interest (COIs) in SLE that could be evaluated using digital clinical measures (DCMs); and (iii) identify target DCMs for their assessment.

**Methods:**

A mixed-methods, multistep approach was deployed for (i) exploring patients’ experience with SLE through a social media listening study and focused group discussions with patients; (ii) mapping patients’ experiences to define MAHs and identify COIs measurable using DCMs; (iii) selecting DCMs for the target COIs; and (iv) identifying types of wearable sensors for measuring COIs in the patients.

**Results:**

Six MAHs related to physical behavior and sleep were identified: difficulty in ambulating, lack of energy, inability to perform activities of daily living, difficulty engaging in sustained walking, inability to perform leisure activities and exercise, and lack of restful sleep. Measurable COIs represented walking (fatigue and pain) and sleep (sleep and pain) characteristics. Five and six DCMs related to stepping behavior and sleep quality, respectively, were identified. Several wearable sensors are available to derive DCMs for physical behavior and sleep; however, patients showed a strong preference for a wrist-worn actigraphy sensor.

**Conclusion:**

We identified DCMs for physical behavior and sleep that are relevant and meaningful to patients with SLE, measurable in a real-world environment with wearable sensors, and have the potential to aid personalized patient care.

## Introduction

Systemic lupus erythematosus (SLE) is a chronic autoimmune disease with heterogeneous clinical manifestations [1, 2]. It is characterized by systemic inflammation and sustained autoantibody production [1, 3]. SLE is approximately nine times more common in women than in men, disproportionately affecting women of childbearing age [4]. The global estimate of SLE incidence in adults ranges 2.2–23.1 per 100,000/year, and the prevalence ranges 30–150 per 100,000 population [1].

SLE is a highly heterogeneous disease comprising various symptoms and unpredictable periods of disease activity that have a substantial impact on the daily lives of patients [5, 6]. The most burdensome symptoms associated with SLE as reported by patients include joint and muscle pain and/or swelling, and fatigue that often affects activities of daily living (ADL), including leisure time and household activities [7–9]. However, studies evaluating specific behavioral adaptations employed by such patients to avoid pain and fatigue are limited. Moreover, several studies have reported that patients with SLE experience poor sleep quality but how sleep is impacted by SLE in patient’s real-world environments remains poorly understood [10–12]. Current tools used to assess patients with SLE are reliant on episodic patient reports and in-clinic assessments. Owing to the chronic nature and high clinical variability of SLE, both within and between individuals, continuous longitudinal monitoring could provide more holistic assessments of patients’ experiences and offer personalized care [13, 14]. The current treatment options for SLE include antimalarials, glucocorticoids, conventional immunosuppressive agents (e.g., methotrexate, azathioprine, mycophenolate, cyclophosphamide), biologics (e.g., rituximab, belimumab, anifrolumab), and voclosporin [5]. Although SLE treatments have improved over recent decades, their limited efficacy and side effects often result in inadequate disease control and prolonged treatments [5, 15, 16]. Therefore, SLE therapies with an improved benefit/risk ratio, which can target the underlying cause of the disease with minimal side effects and improve the health-related quality of life (HRQoL) of patients, are urgently required [16].

Wearable and connected technologies provide a significant opportunity to support patient-focused drug development by enabling the capture of novel, real-world digital clinical measures (DCMs) that reflect a patient’s lived experience and are clinically valid and sensitive to treatment benefits [17]. Owing to the heterogeneous nature of SLE, digital health technologies (e.g., wearable sensors and other connected technologies) capable of capturing long-term disease manifestations and progression are of particular interest and provide an opportunity to develop a new class of validated outcome measures that are meaningful to patients and clinically relevant. For example, in clinical studies for ambulant patients living with Duchenne muscular dystrophy, the European Medicines Agency has recently qualified the stride velocity 95th centile as a primary endpoint [18].

In line with US Food and Drug Administration (FDA) guidance on patient-focused drug development, the Digital Medicine Society has outlined key steps to be followed in the selection and validation of DCMs; these include the identification of meaningful aspects of health (MAHs) and concepts of interest (COIs), selection of relevant digital measures, analytical and clinical validation of measures, and deployment and use of these measures in clinical care/research [19, 20]. For novel, real-world DCMs to realize their full potential and improve outcomes for patients with SLE by providing objective and reliable SLE-related data, it is crucial to identify key MAHs and COIs in SLE. The purpose of this study was to investigate patients’ experience with SLE, including symptoms, disease impact, and treatment, with the aim to identify potential MAHs and COIs as a first step to develop target DCMs for assessment in SLE.

## Methods

**Fig. 1:**
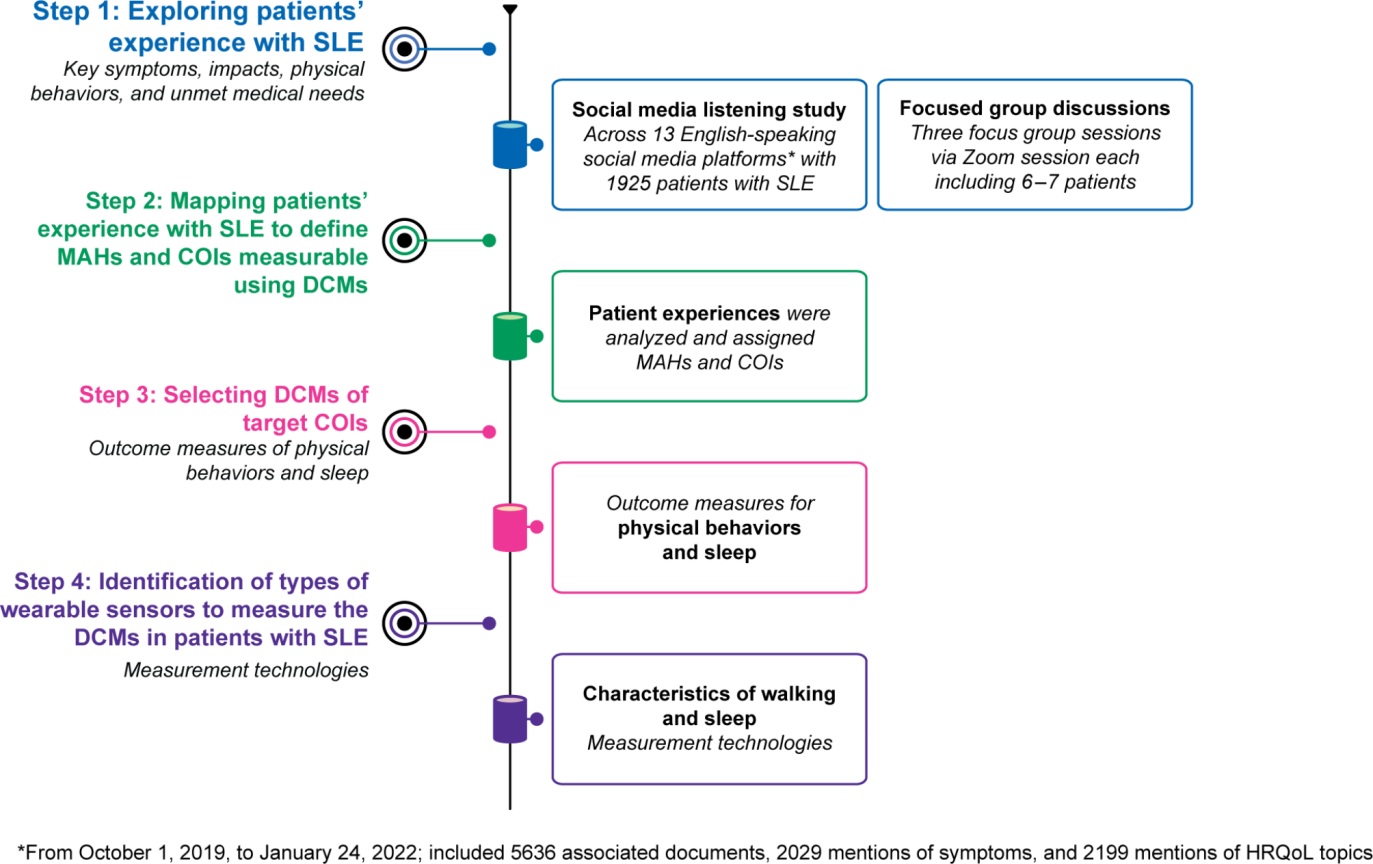
Mixed-methods multistep approach for identifying MAHs and COIs for assessment using DCMs in SLE. *Abbreviations COIs* concepts of interest, *CPIW* Centrepoint Insight Watch, *DCMs* digital clinical measures, *HRQoL* health-related quality of life, *MAHs* meaningful aspects of health, *SLE* systemic lupus erythematosus

We followed a mixed-methods multistep approach to develop a conceptual framework for the identification of DCMs: step (1) exploring patients’ experience with SLE; step (2) mapping patients’ experience with SLE to define MAHs and COIs measurable using DCMs; step (3) selecting DCMs for target COIs; and step (4) identification of types of wearable sensors to measure the DCMs in patients with SLE (Fig. 1) [19].

### Step 1: exploring patients’ experiences with SLE

Patients’ experience with SLE, including key symptoms, disease impact on physical behaviors, and unmet medical needs in symptom monitoring and overall treatment, was evaluated using a social media listening study and focused group discussions (FGDs).

#### Social media listening study

A retrospective social media listening study of 13 English-language social media platforms was conducted between October 1, 2019 and January 24, 2022, to investigate the disease burden, treatment experiences of patients with SLE, and their impacts on HRQoL [21]. SLE-related keywords were defined and revised by human domain experts, individuals with specialized knowledge and expertise in the field, using the application programming interface of a third-party social media data provider, Social Gist (https://socialgist.com/). The keywords used for the search included: “lupus,” “lupus” AND “systemic” AND “erythematosus” AND “autoimmune” AND “disease,” and “SLE”. Social media posts were imported into Pharos^®^, a proprietary data visualization and analysis platform from Semalytix GmbH (Bielefeld, Germany) and natural language processing (NLP) techniques were used to filter and aggregate relevant patient-reported content and algorithmically coded patient experience themes, based on the World Health Organization Quality of Life instrument (WHOQOL)-100 [22]. Non-NLP analysis included descriptive statistics of concepts and thematic content analysis to describe patient experiences.

#### Focused group discussions

The Center for Information and Study on Clinical Research Participation (CISCRP), an independent nonprofit organization, collaborated with the Lupus Foundation of America to help identify patients with SLE for FGDs in their Research Accelerated by You (RAY^®^, Pro#00020515), a lupus data platform with self-reported data from patients with lupus. Patients aged between 18 and 75 years who had a confirmed diagnosis of SLE by a physician, moderate to severe disease activity as determined by the clinician’s judgment or standardized assessments, and received oral/parental corticosteroids, oral antimalarials or immunosuppressants for the treatment of SLE within the last 30 days were eligible. However, patients receiving dialysis treatment (current or past) and those who underwent organ transplant procedures were excluded. All participants provided written informed consent before their participation in the FGDs. The sessions were led by a single moderator who used a structured discussion guide and invited all patients in each focused group to contribute to the discussion. The sessions were recorded, and anonymized transcripts were available for analysis.

The first and second FGD solicited feedback on patient experiences of living with SLE to better understand the symptom management, day-to-day changes in disease symptoms and daily routine of patients. The goal of these FGDs was to identify the measurable components of behavior that were impacted by SLE. A discussion guide included questions to determine how aspects of daily life were impacted by common symptoms of SLE (fatigue, pain) and asked participants to describe how their sleep and aspects of physical activity were impacted by SLE. When appropriate, additional probes were used to capture more detailed information on the attributes of physical behaviors, such as frequency, intensity and duration, most impacted in response to symptom and aspects of SLE (e.g., are there behaviors you perform less frequently when you are experiencing fatigue; does intensity of behaviors change when you are experiencing fatigue; do you perform behaviors for different durations when you are experiencing fatigue).

Given the limited data available describing the feasibility and acceptability of using digital health technology (DHTs) in patients with SLE, the third FGD explored patient perspectives towards the use of different wearable technologies to monitor their SLE symptoms and daily behavioral outcomes. Representative images and descriptions of wearable sensors that could capture measures of physical behavior and sleep were presented to participants. Two different actigraphy sensors that could capture physical behaviors and sleep behaviors were presented to participants: a wrist-worn actigraphy sensor and a thigh-worn actigraphy sensor. In addition to the actigraphy sensors, two additional form factors to measure sleep behaviors were presented to participants: a muti-modal sensor containing a wrist band and finger worn pulse oximeter, and a headband sensor. For each wearable sensor, participants were asked about their overall thoughts on the device (anticipated ease of use/comfort, concerns, likes/dislikes), and the likelihood that technology would fit into their daily routine.

### Step 2: mapping patients’ experience with SLE to define MAHs and COIs measurable using DCMs

**Fig. 2:**
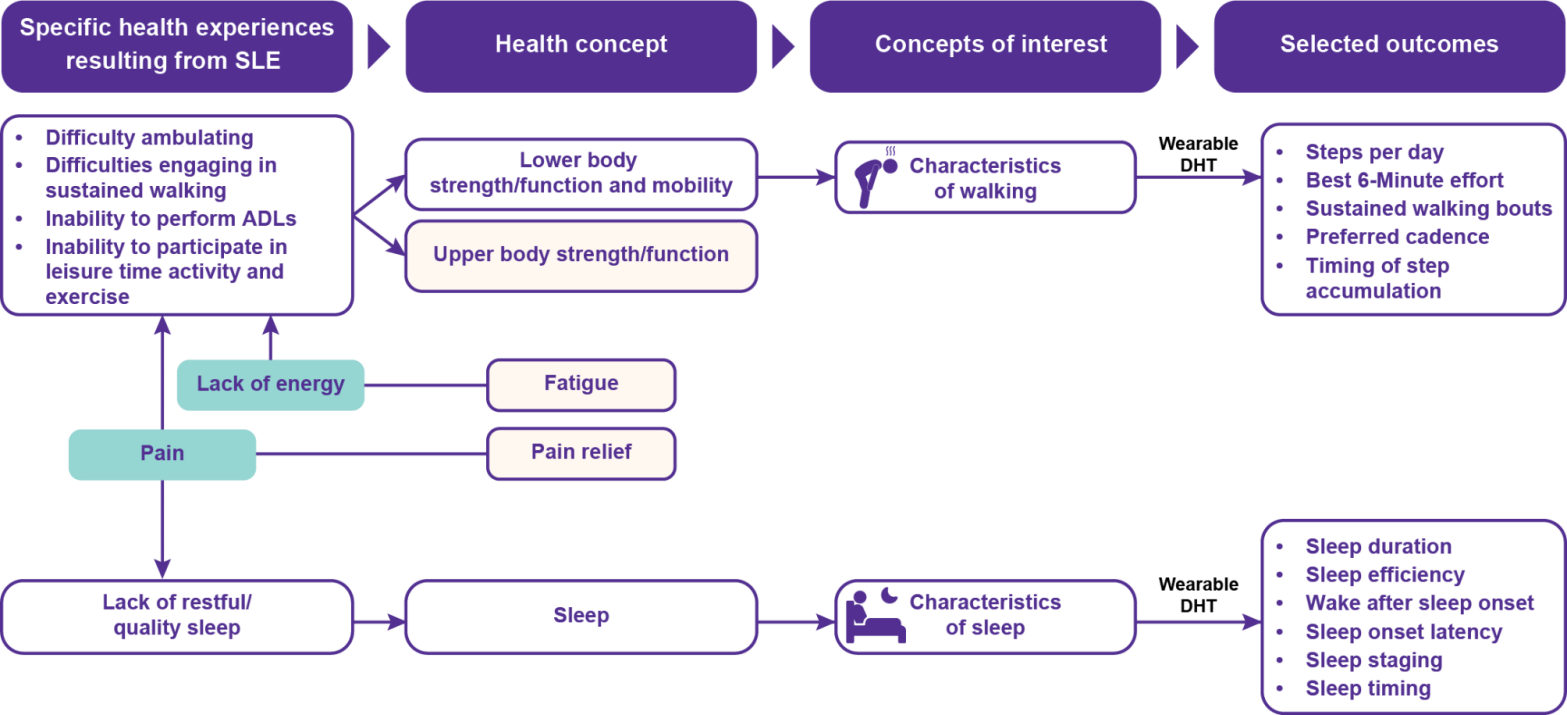
Conceptual framework of SLE digital clinical measures. Figure illustrates the conceptual framework linking specific health experiences resulting from SLE reported by patients to outcomes that can be measured with a wearable DHT. Patients reported difficulties in performing a range of physical behaviors (top group of health experiences in the figure). Such behaviors map to health concepts of lower and upper body strength/function and mobility. As indicated in the figure, lower body strength/function and mobility map to the COI of characteristics of walking and can be measured with wearable DHTs. Upper body strength/function is more complicated to measure with a wearable DHT and as such no COI or outcomes were identified. Represented by the blue arrows in the figure, lack of energy and pain are health experiences that patients reported to be important and impact their ability to perform a range of physical behaviors and sleep. Pain and fatigue are inherently subjective and assessing such health concepts is not feasible with a wearable DHT; therefore, no concept of interest or outcomes were defined for pain and fatigue. *Abbreviations ADL* activities of daily living, *COIs* concepts of interest, *DHT* digital health technology, *HRQoL* health-related quality of life, *SLE* systemic lupus erythematosus

Patients’ symptom-related experiences were summarized using information obtained from social media listening study and FGDs. To develop a conceptual framework, common health experiences reported by patients were first grouped into MAHs. The underlying health concepts related to MAHs were then mapped. A selection of DCMs to measure the concepts were finally identified which could be captured with wearable sensors in patients with SLE (Fig. 2; Table 1).

**Table 1:** MAHs and target COIs identified from the patient experience data

Focused group discussions	Social media listening study	MAHs	Health concepts
Nearly all patients struggled with daily fatigue, describing it as extreme tiredness that was not alleviated by sleep. Most patients found their fatigue to be constant, that occurred on both good days and bad days, in varied severity. Patients described fatigue as both physical and mental. *“For me*,* it is like I am a balloon*,* and someone has poked a hole in me*,* and the air is slowly going out. As the fatigue sets in*,* my air goes out. I have no more to give. Just no more energy.”*	Many patients reported extreme levels of daytime tiredness that impacted their abilities to perform ADL and leisure time tasks. *“I am so exhausted that I cannot shower*,* make meals*,* leave my house*,* or do anything other than lie in bed and read or sleep*,* all day/all night.”*	Lack of energy	Fatigue, ADL
All patients reported experiencing pain to some extent at various body locations and that it had the biggest impact on their lives affecting their ability to perform walking activities. *“For me a good quality of life is being able to keep up with them – and participate in the things that they want to do*,* whether it’s hiking or biking or walking.“* *“But for me*,* it’s constant. It’s always something. It’s my hands*,* my wrists*,* my feet*,* my hips when I walk. That definitely is – if you could take away the things that interfere the most with the happiness in your life*,* for me it’s the constant pain that you’re always in.”*	The most burdensome symptoms reported were all pain-related symptoms. *“My occasional inability to make it up a set of stairs*,* or becoming a poor-performing robot who moves very*,* very slowly and cannot operate any small digits (fingers*,* toes*,* etc.).”*	Difficulty ambulating, pain relief	Pain, ADL, lower body strength/function and mobility
Regular movement was important to patients to help them manage SLE symptoms; they reported feeling much worse if they skipped their activity. *“My body is really aching and throbbing right now. I woke up this morning and…it was tough to actually walk my dog down the steps to let her out.”* *“[my level of activity is] Low to maybe moderate activity on a daily basis. I would like to be able to go for a walk or exercise more…[SLE] really limited my ability to do activities that I used to enjoy.”*	46% of patients who used the discussed nondrug therapies reported exercise (specifically walking) as a strategy to manage SLE symptoms, including fatigue and pain. *“For me*,* stretching helps and swimming*,* and even just walking in a pool.”*	Difficulties engaging in physical activities such as walking, pain relief	Fatigue, pain, lower body strength/function and mobility
Patients reported that their ability to perform ADL requiring prolonged periods of walking and/or standing (e.g., grocery shopping, cooking, vacuuming, dishes) was limited by their fatigue and pain. *“I place my grocery orders online. I pull up to the grocery store*,* pop my trunk*,* they put them in the trunk*,* and I drive away. Because walking around a grocery store for 30/40 minutes just hurts.”* *“I have so much myofascial pains. It’s really limited my ability to do activities that I used to enjoy. Like cooking – standing in the kitchen for too long hurts.”* *“Believe it or not*,* vacuuming can make me feel like I’m having a heart attack.”* *“Standing washing dishes for even – I feel like after 10 min*,* my hips feel really heavy – lower back. My feet feel really heavy.”* Some patients reported experiencing pain in their hands which limits their strength and ability to do ADL such as opening jars etc. *“I can’t open up jars of things anymore*,* because my hands have – they either hurt or they’ve lost strength.”*	Patients reported difficulty completing ADL when experiencing pain or fatigue. *“Never in my life would I have known that cleaning my house would be a problem. […] Sometimes the pain is so severe in my muscles that I can hardly walk.”*	Difficulties engaging in physical activities such as walking, pain relief	Fatigue, pain, lower body strength/function and mobility
Most patients tried to maintain some structured physical activity, noting its importance to overall health and the benefits of managing symptoms of pain and fatigue. *“I think it is a matter of being able to do things. There are events I would like to go to*,* but if they require too much walking or too much standing*,* I cannot do them. I think it is just being able to have some sort of quality of life to be able to do the things I used to be able to do*,* knowing full well that I cannot do everything that I used to be able to do.”* Many patients reported that their level of physical activity was drastically different depending on the day. On “bad days”, they severely limited movement in any capacity and most remained in bed until their symptoms subsided. *“There are days when I wake up and I feel pretty good*,* and it’s not a bad day. And then there are days when I wake up and I immediately know as soon as my feet hit the floor that it’s going to be a struggle.”* *“I feel fatigue was a real issue for me in my early stages of lupus*,* where I didn’t have the energy and you just feel like your tank is on ‘E.’ You don’t have the energy to do anything.”* When experiencing a “good day”, patients revealed they were compelled to take advantage of their improved health. Most patients therefore engaged in more physical activities, but often found that pushing themselves more than normal on those good days would lead to bad days soon after. *“When you feel good*,* you try to take advantage of it and pretty much do anything you can*,* and that can be counterproductive. Because it could sideline you for days later or make your joints more inflamed with more inflammation.”* *“When there are good days*,* I like to try and go do things*,* as long as I don’t overdo it. Because I find for me*,* if I do too much physically*,* I pay for it for the next few days and have a tough couple of days following.”*	Some patients noted that they were unable to participate in structured physical activity (e.g., running) because of pain and fatigue. *“But now the joint pain is back so badly*,* and I cannot sleep at all and feel so fatigued during the day. I feel too tired to join my run group this evening…”*	Inability to participate in leisure activities and exercise, difficulties engaging in physical activities such as walking, pain relief	Fatigue, pain, lower body strength/function and mobility
Patients revealed that pain had the biggest impact on their quality of sleep and ability to fall asleep as well as maintain sleep. *“When my hips are hurting*,* if I lay on one side too long*,* I get woken up from the pain. And then it hurts to move to the other side*,* but I have to because I can’t go back to sleep because it hurts.”*	Patients frequently reported sleep problems, including insomnia. *“I have had sleeping issues for over 2 years now and somedays I cannot sleep for 48 hours straight.”*	Lack of restful/quality sleep, pain relief	Sleep, pain

*Abbreviations ADL* activities of daily living, *COIs* concept of interest, *MAHs* meaningful aspects of health, *SLE* systemic lupus erythematosus

### Step 3: selecting DCMs for target COIs

Based on FGDs, the identified COIs were evaluated for the various aspects of behavior that could be measured with DCMs using available digital health technologies. The outcome measures selected for physical behavior and sleep are shown in Fig. 2.

### Step 4: identification of types of wearable sensors to measure the DCMs in patients with SLE

Numerous digital health technologies/wearable sensors are available to derive DCMs for physical behavior and sleep. The measurement technologies that have been previously used in clinical research and have demonstrated ability to collect real-world data with the rigor required in regulated clinical trials were selected. As described earlier, patients in the third FGD were presented with an overview of wearable sensors types that can capture measures of physical behavior and sleep, and asked to provide their feedback on the technology.

## Results

### Step 1: exploring patients’ experiences with SLE

#### Social media listening study

A total of 1925 self-identified patients with SLE using social media outlets were included in this retrospective social media listening study [21]. In total, 5636 posts were examined, which included 2023 mentions of “symptoms” and 2199 mentions of “HRQoL” topics. Pain (30.1%), fatigue (19.2%), and rash (11.8%) were reported as the most burdensome symptoms affecting ADL [21]. Fatigue showed a broader distribution of impairments over several quality of life (QoL) facets, whereas pain primarily affected mobility (*n* = 38 posts), recreation and leisure (*n* = 16 posts), and sleep and rest (*n* = 9 posts). Considering individual QoL facets separately, mobility, cognitive capabilities, and recreation and leisure were most frequently reported by patients as being impaired, with fatigue and pain being the most prominent drivers of impairment in each of these facets and affecting overall QoL.

While hydroxychloroquine (34.4%), prednisone (20.6%), and methotrexate (19.3%) were the most frequently reported drug therapies, exercise was the most frequently reported non-pharmacological therapy (46.1%).

#### Focused group discussions

The CISCRP conducted three FGDs (each lasting 2 h) via the Zoom platform on March 3, 2022 (*N* = 6 [5 females, 1 male]; 2 African Americans, 1 African American and Hispanic, and 3 Caucasians), April 5, 2022 (*N* = 7 [5 females, 2 male]; 4 African Americans, 2 Caucasians, and 1 Pacific Islander), and August 2, 2022 (*N* = 6 [5 females, 1 male]; 3 African Americans, 2 Caucasians, and 1 Pacific Islander), and were led by a single moderator. The moderator led each focus group using a structured discussion guide and invited all patients in each focused group to contribute to the discussion.

The first FGD evaluating the patient experience concluded that lack of energy and fatigue are common for patients with SLE and represent symptoms that cannot be resolved by sleep. In the patient’s own words, they described this feeling as *“I could sleep and sleep and sleep*,* and it does not resolve the fatigue.”* In the second FGD, considerable variability in tiredness and fatigue was reported within and between patients. Many patients reported that their tiredness persisted throughout the day and culminated in a complete lack of energy and inability to perform leisure activities and ADLs. One of the patients described the feeling as *“There are times when I will spend an entire weekend literally just sitting or lying on the couch*,* binge-watching TV*,* or just so tired that I just sort of lay there and not even watch anything because I cannot stay focused on anything long enough to keep my mind occupied.”*

The patients in the third FGD reported that they frequently tracked their lifestyle behaviors (physical activity, nutrition) and noted worsening symptoms and health events using applications on their phone or online patient portals. Tracking behavior and symptoms helped patients with self-management and was beneficial for facilitating the clinical visits.

### Step 2: mapping patients’ experience with SLE to define MAHs and COIs measurable using DCMs

Through FGDs, six MAHs related to physical behavior and sleep were identified (Fig. 2; Table 1). (i) **Lack of energy**: Lack of energy and fatigue are common in patients with SLE, occasionally resulting in patients staying in bed all day, although there is considerable variation within and between patients. (ii) **Difficulty ambulating**: Walking can be performed at low intensity and is meaningful to patients. Joint stiffness and pain are some of the most common and distressing symptoms for patients with SLE, resulting in difficulty moving within their real-world environments, necessitating modifications to movements (e.g., walking with a limp, move more slowly), reducing the total amount of movement (e.g., avoid stairs, limit walking), and increasing the sitting time or limiting movements. (iii) **Difficulties with sustained walking**: Some patients often complete only short distances owing to pain and fatigue and require frequent rest breaks, whereas others attempt to include bouts of walking in their daily routine to manage SLE symptoms despite underlying fatigue and pain. (iv) **Inability to perform ADLs**: Fatigue limits the ability of patients with SLE to perform ADLs, resulting in patients avoiding certain activities, reducing the frequency of those activities, or modifying the way they undertake them (e.g., using online delivery services, taking frequent rest periods). (v) **Inability to participate in leisure activity and exercise**: Symptoms of fatigue and pain can limit a patient’s ability and motivation to participate in leisure activities and structured exercise. Patients with SLE recognize the importance of physical movement in managing the symptoms and prognosis of SLE, and many report performing structured exercises (walking, yoga, cycling, etc.) despite experiencing significant fatigue or pain. Many patients report that they feel worse when they skip an exercise session. (vi) **Lack of restful sleep**: Sleep disturbances are often associated with SLE and represent a major determinant of QoL. Broadly, patients report difficulty falling asleep, maintaining sleep, and having sufficient sleep to feel well-rested upon waking.

The identified MAHs were mapped to measurable COIs representing walking and sleep characteristics, which were in turn influenced by other COIs representing common symptoms frequently experienced by patients with SLE, including pain, fatigue, and sleep (Fig. 2; Table 1).

### Step 3: selecting DCMs for target measurable COIs

The physical behaviors in SLE are complex, and walking behavior represents one aspect of physical functioning that is commonly impacted and can be reliably measured with wearable actigraphy sensors. Based on the patients’ experiences of SLE and MAHs defined, five measures related to stepping behaviors (steps per day, best 6-min effort [23], sustained walking bouts, preferred cadence, and timing of step accumulation) and six measures related to sleep quality (sleep duration, sleep efficiency, sleep onset latency, sleep staging, wake after sleep onset, and sleep timing) were identified as potentially relevant and feasible in SLE (Fig. 2; Table 2). The key challenges and measurement considerations to justify the use of DCMs for patients with SLE are presented in Table 3. These outcome measures reflect aspects of health that are quantifiable and meaningful to patients. The rationale for selection of each DCM of physical behavior and sleep is summarized in Table 3.

**Table 2:** Physical behavior and sleep outcome measures and rationale for their selection

Outcome measure *(unit)*	Rationale for selection of DCMs
Characteristics of walking behavior	
Steps per day *(steps/day)*	Although walking is the preferred form of activity for patients with SLE, their ability to walk can be limited by the symptoms of the disease.
	Step recommendations are frequently used in public health messaging because they are easily understood and relatively easy to measure with simple tools. Steps per day can provide a high-level summary of an individual’s level of physical activity.
Best 6-min effort (B6ME) *(number of steps*,* steps/min)*	Patients with SLE report difficulties walking because of the fatigue and pain they experience.
	B6ME identifies the 6-min period during which patients exert their best effort daily. Several stepping performance metrics can be derived during this 6-min window to summarize a patients’ maximal real-world walking ability (e.g., number of steps, cadence).
Sustained walking bouts *(min)*	Patient reports indicate that they avoid continuous activity and/or break up long walking bouts.
	Capturing the time spent in sustained walking bouts of varying durations would contextualize how patients with SLE perform stepping behavior.
Preferred cadence per day *(steps/day)*	Individuals tend to step at a consistent rate (steps per min). Patients with SLE report walking at slower speeds because of fatigue or joint pain they experience.
	Preferred cadence measures the median cadence of all walking bouts of >10 s in duration. Given that patients with SLE may adjust how they walk when they are feeling fatigue or pain (i.e., reduce their walking speeds), preferred cadence would provide an assessment of real-world walking performance.
Timing of step accumulation *(time of day)*	Patients with SLE frequently lack energy during the day, which ultimately results in the need to rest and stop moving at the end of the day, although the time at which this occurs is highly variable both within and between patients.
	Investigating the timing of step accumulation relative to the time of waking (e.g., the time at which 50% of steps are accumulated with a day) can provide insights into the timing of fatigue and capture shifts in the way patients perform stepping behaviors.
Characteristics of sleep	
Sleep duration *(min)*	Most patients with SLE report difficulty sleeping and staying asleep throughout the night for a range of reasons, including pain.
	Sleep duration is a straightforward metric that captures the time someone spends asleep at night and is incorporated into public health guidelines.
Sleep efficiency *(percentage)*	Frequent awakenings and poor sleep efficiency are common in patients with SLE.
	Sleep efficiency measures the proportion of time an individual is in bed and asleep and can provide an objective assessment of sleep quality.
Wake after sleep onset (WASO) *(min)*	Awakenings during sleep represent another metric that reveals insights into sleep quality. WASO summarizes the total time an individual is awake after falling asleep and provides an objective measure of another aspect of sleep quality.
Sleep onset latency *(min)*	Patients with SLE also report that they experience difficulty falling asleep and can spend extended periods of time trying to find a body position that does not induce pain.
	Sleep onset latency captures the time it takes for an individual to fall asleep after going to bed and represents another way to capture sleep quality.
Sleep staging *(min)*	Patients with SLE report that they rarely wake up feeling refreshed and are plagued by frequent awakenings that alter sleep cycles and prevent them from entering deeper sleep stages that are critical for restoration, memory, and information consolidation. Sleep staging provides additional context to the quality of sleep beyond sleep duration alone.
Sleep timing *(time of day)*	SLE symptoms can significantly impact sleep timing (when a patient goes to bed and wakes up). Many patients experience extreme fatigue during the day, causing them to go to bed very early (e.g., before having dinner with family). Others report having to stay in bed for long periods of time, which causes them to miss activities they want to partake in (e.g., getting kids ready for school).

*Abbreviations DCMs* digital clinical measures, *SLE* systemic lupus erythematosus, *WASO* wake after sleep onset

**Table 3:** Rationale for recommendations of physical behavior and sleep outcomes—challenges (o) and measurement considerations (❖) across evidence types to justify the use of DCMs in patients with SLE

Evidence type	Physical behavior outcomes	Sleep outcomes
Meaningful patient experiences (MAHs)	o Patient reports indicate that the SLE disease experience is highly variable within and between patients o Both SLE-related and non-SLE-related factors influence an individual’s decision to participate in physical behavior (e.g., bad weather and SLE-related fatigue could both influence whether a patient decides to go for a walk outside) ❖ The high within- and between-patient variability of physical behaviors necessitates (i) prolonged and continuous measurement of physical behavior and (ii) that changes in physical behaviors be expressed relative to a patient’s baseline	o Sleep problems are consistently observed in patients with SLE, and patients frequently report poor sleep regardless of total sleep duration ❖ Capturing sleep outcomes beyond “total duration” is essential in patients with SLE
Measurable in a patient’s real-world with wearable sensor	o Wrist actigraphy sensors conducive to long-term measurement can continuously and passively capture physical behavior ❖ Measurable outcomes of physical behavior using wrist actigraphy are limited to characteristics of walking	o Low burden actigraphy sensors are insufficient to capture aspects of sleep beyond “total duration” ❖ Measurement tools that capture alternative or additional signals to wrist movement are strongly needed
Clinically relevant in SLE	o Owing to the heterogeneity of disease manifestations, there is currently a lack of clinical validity evidence for physical behavior outcomes in patients with SLE ❖ Physical behavior outcomes can be positioned as exploratory assessment in SLE clinical trials	o Sleep is clinically relevant for nearly every health outcome across multiple therapeutic areas ❖ Given the extensive clinical validity evidence of sleep across therapeutic areas, there is strong scientific rationale to position sleep as a secondary assessment in SLE clinical trials

*Abbreviations COIs* concepts of interest, *DCMs* digital clinical measures, *MAHs* meaningful aspects of health, *SLE* systemic lupus erythematosus

### Step 4: identification of types of wearable sensors to measure DCMs in patients with SLE

The third FGD explored patient attitudes towards different wearable sensors, and after reviewing all four types of measurement sensors presented, top patient preference was for the wrist-worn actigraphy sensor, followed by the thigh-worn actigraphy sensor. The headband type sensor and multi-modal wrist-worn sensor were least preferred among the group. In comparison to the other technologies, patients were most comfortable and familiar with the wrist-worn actigraphy sensor to monitor their levels of activity and sleep characteristics, as many currently wore smartwatches. Patients also felt this device would be the most comfortable and the easiest to adhere to for extended periods of time, particularly if experiencing a bad day or flare. In the patient’s own words, they described this feeling as *“I would totally do this just because it seems fairly simple and easy. Just slap it on my wrist.”* For many, the thigh-worn actigraphy sensor was viewed as minimally disruptive and, therefore, ranked second by nearly all patients. Many patients showed their willingness to use the device at least for limited periods of time as the sensor would not be impacted by a good day, bad day or flare, given the passive nature of the technology.

In general, patients were most hesitant to the sleep monitors. As nearly all patients experienced significant sleep difficulties; they felt any disruption or adjustment to their current sleep regimen would negatively impact the already minimal amount of sleep they currently achieve. Patients were also very concerned about the number of wires and potential for entanglement associated with the multi-modal wrist-worn sensor. Some also spoke to how they don’t like wearing anything on their head while sleeping, therefore ranking the headband sensor low compared to other sensor types. One patient described this as *“Sure*,* I ranked mine as the wrist-worn as the first and the thigh-worn as second. The hairband*,* third*,* and the multi-modal*,* fourth. Pretty much I’m thinking of comfortability*,* the easy access*,* easy to actually wear. The watch*,* we can put it on*,* and leave it on*,* and it’s waterproof.”*

## Discussion

Digital health technologies, such as wearable sensors and other connected technologies, are driving a digital revolution in healthcare and clinical research [24–30]. Such tools can be deployed remotely, passively, and continuously for longitudinal monitoring of the disease experience of patients living with SLE. The use of digital health technologies capable of capturing long-term disease manifestations and progression in diseases such as SLE, characterized by variable periods of disease activity, can aid patient-focused drug development, and facilitate personalized care [17]. Although physical behavior and sleep outcomes are important indicators of health in patients with SLE, most patient-reported data fail to provide the level of detail necessary to identify specific outcomes measures that are both meaningful and measurable in patients with SLE. Currently, there is limited evidence linking physical behavior and sleep outcome measures to clinically meaningful outcomes in patients with SLE. However, there is strong scientific justification to support their meaningfulness and to associate physical behavior and sleep outcomes with important indicators of health in this population. Gallop et al. conducted semi-structured interviews of patients with SLE (*N* = 72) and proposed a conceptual model based on patient experience, highlighting the impact of SLE on patients’ HRQoL, including their ability to perform ADL [31]. Similarly, Cleanthous et al. proposed a conceptual model of SLE fatigue using semi-structured interviews (*N* = 22) and reported that patients experience three overarching domains of fatigue: physical, mental, and cognitive, and susceptibility to fatigue [32]. However, these studies do not address which of the patient experiences can be measured in the real-world setting using DCMs. To the best of our knowledge, this is the first study to provide a conceptual framework for identifying MAHs and COIs for assessment using DCMs that are meaningful to patients with SLE. Additionally, this is the first study which explored the patient attitudes towards various wearable sensors and the acceptance of technology in this specific patient population.

In patients with SLE, real-world measures of physical behavior and sleep pose unique challenges that require careful consideration and necessitate contextualizing continuous wearable sensor data with patient-reported outcomes (e.g., identifying a “good day” or a “bad day”) which can account for SLE-dependent and independent factors affecting patient behaviors to improve their interpretability. Patients with SLE often experience periods of disease activity, resulting in significant disease burden [33, 34]. Moreover, fatigue, pain, difficulty in moving, and sleep problems are common to all patients with SLE, although a high degree of variability is observed within and between patients in response to these symptoms [33, 34]. Depending on the individual, physical behavior may remain unchanged or be reduced or abandoned. To capture oscillations in disease activity and manifestation across multiple patients, long follow-up periods are essential and could range from several months to over a year depending on the patient population and disease burdens or flares.

The decision of patients with SLE to participate in physical activities can be impacted by both SLE-related (e.g., pain, fatigue, flares, other SLE manifestations) and non-SLE-related factors (e.g., pain, fatigue, work/disability status, weather, lack of time), which may prevent or encourage physical activity [35–37]. For example, fatigue may negatively impact motivation levels and result in low step counts. Additionally, patients can experience pain and fatigue, which may limit their decision to participate in physical activity for reasons other than SLE. Although some patients report reducing their activity levels on a “bad day,” this is not consistent within or between patients. Therefore, it is difficult to anticipate which physical behavior patterns determine whether a patient is experiencing a “good day” or a “bad day” or how these real-world behaviors might change as a treatment benefit. Furthermore, in real-world environments, it is impossible to completely isolate these factors from each other. However, contextualization of wearable sensor data with patient-reported outcomes (e.g., identifying a good or bad day) and comprehensive characterizations of patient demographics (e.g., age, work/disability status, family/parental responsibilities) can help clarify these factors. Similar to physical behaviors, several factors independent of SLE can cause sleep disruptions, including room temperature [38], light exposure [39], noise [40], or psychosocial stress [41], and contextualizing wearable sensor data with patient-reported outcomes that capture these factors can improve interpretability of the data.

The identification of MAHs and COIs in SLE underpinning our work was based on a large body of evidence derived from the patient experience data from multiple sources. We applied best practices and patient-centric approaches endorsed by the FDA to identify MAHs in patients with SLE, determine COIs, and define outcomes that are measurable with wearable sensors [17]. DCMs can play a significant role in supporting future patient-focused drug development in SLE as potential clinical trial outcome measures; however, capturing the experience of patients effectively and accurately using wearable sensors is complicated. Although there is scientific rationale to support the clinical meaningfulness of physical behavior and sleep outcome measures, the evidence of clinical validity is currently limited. Therefore, a key next step for DCMs for physical behavior and sleep is to accumulate analytical and clinical validation evidence for patients with SLE. Moreover, acceptance from regulatory agencies for specific DCMs as fit-for-purpose outcome measures in populations with SLE is crucial. Communicating with regulators and discussing the use of digital health technologies to capture MAHs is a crucial step in developing DCMs and obtaining regulatory approvals.

## Limitations

The social media listening study utilized publicly available social media data to evaluate patient’s experience with SLE and has several limitations [21]. First, there are concerns about the authenticity of the information obtained from social media platforms, as posts may not always reflect verified medical diagnoses or experiences. Additionally, patients may self-diagnose or self-report their disease experiences without formal medical confirmation, which could lead to potential inaccuracies in the data collected. Since some patients chose not to share their profiles publicly, they were consequently excluded from the study. Second, the dataset might include duplicate profiles, as some users may have been active on multiple social media platforms. Third, the study focused solely on English-speaking countries and younger populations. The data analyzed in this study were obtained from publicly available sources and appropriate steps were taken to anonymize the posts. In addition, a strict general data protection regulation compliant process was adopted to ensure personal data protection. The FGDs used to gather patient’s experience with SLE had a relatively small sample size and relied solely on descriptive analysis. While descriptive analysis offers a comprehensive summary of patients’ experience, it may have limitations in providing in-depth insights to contextualize the data.

## Conclusions

This study identified DCMs for physical behavior and sleep that are meaningful to patients with SLE, and measurable with wearable sensors in a patient’s real-world environment. The use of digital health technologies in capturing the long-term manifestation and progression of variable diseases such as SLE can aid patient-focused drug development and lead to personalized care and improved outcomes for patients with SLE.

## Data Availability

Any requests for data by qualified scientific and medical researchers for legitimate research purposes will be subject to the healthcare business of Merck KGaA, Darmstadt, Germany’s (CrossRef Funder ID: 10.13039/100009945). Data Sharing Policy: All requests should be submitted in writing to the healthcare business of Merck KGaA, Darmstadt, Germany’s data sharing portal (https://www.emdgroup.com/en/research/our-approach-to-research-and-development/healthcare/clinical-trials/commitment-responsible-data-sharing.html). When the healthcare business of Merck KGaA, Darmstadt, Germany has a co-research, co-development, or co-marketing or co-promotion agreement, or when the product has been outlicensed, the responsibility for disclosure might be dependent on the agreement between parties. Under these circumstances, the healthcare business of Merck KGaA, Darmstadt, Germany will endeavor to gain agreement to share data in response to requests.
